# Fatal Outcome Following Polysubstance Use: A Case Report of Rhabdomyolysis, Acute Kidney Injury, and Deep Vein Thrombosis

**DOI:** 10.3390/toxics14040273

**Published:** 2026-03-25

**Authors:** Stanila Stoeva-Grigorova, Ivanesa Yarabanova, Ivelina Panayotova, Maya Radeva-Ilieva, Georgi Bonchev, Milan Tsekov, Delyan Ivanov, Mario Milkov, Simeon Marinov, Petko Marinov, Snezha Zlateva

**Affiliations:** 1 Department of Pharmacology, Toxicology and Pharmacotherapy, Faculty of Pharmacy, Medical University of Varna, 9000 Varna, Bulgaria; maya.radeva@mu-varna.bg (M.R.-I.); petko.marinov@mu-varna.bg (P.M.); snezha.zlateva@mu-varna.bg (S.Z.); 2 Clinical Toxicology Department, Naval Hospital, 9000 Varna, Bulgaria; ivanesa_98@abv.bg; 3 Laboratory of Analytical Toxicology, Naval Hospital, 9000 Varna, Bulgaria; stefanova_vma@abv.bg (I.P.); georgi.bontchev@gmail.com (G.B.); 4 Department of Vascular Surgery, Naval Hospital, 9000 Varna, Bulgaria; milantsekov74@gmail.com; 5 Department of Psychiatry and Medical Psychology, Faculty of Medicine, Medical University of Varna, 9000 Varna, Bulgaria; 6 Department of Dental Materials Science and Prosthetic Dental Medicine, Faculty of Dental Medicine, Medical University of Varna, 9000 Varna, Bulgaria; mario.milkov@mu-varna.bg; 7 Department of Urology, Faculty of Medicine, Medical University of Varna, 9000 Varna, Bulgaria; dr.marinov.simeon95@gmail.com

**Keywords:** polysubstance abuse, fentanyl, cocaine, methamphetamine, cannabis, rhabdomyolysis, acute kidney injury, thrombosis, hemodialysis, clinical toxicology

## Abstract

**Background:** Polysubstance use, particularly the combination of opioids and stimulants, represents a growing public health concern due to its high risk of severe multisystem complications and mortality. Here, we present a case illustrating the lethal synergy of opioid–stimulant co-use. **Methods:** A 37-year-old male with chronic Hepatitis C and documented polysubstance use reported recent use of fentanyl, cocaine, methamphetamine, and cannabis. He presented with generalized weakness, left lower limb pain, tense edema, and anuria. Clinical assessment included monitoring of vital signs, physical examination, capillary blood gas analysis, extended laboratory panels (muscle and cardiac enzymes, electrolytes, and coagulation parameters), urinalysis, and Doppler imaging. Management over five days included intravenous hydration, diuretics, urinary alkalinization, electrolyte correction, anticoagulation, metabolic and vitamin therapy, hemodialysis, and comprehensive supportive care. **Results:** Laboratory evaluation revealed massive rhabdomyolysis (peak CK 161,050 U/L), severe hyperkalemia (K^+^ 8.4 mmol/L), metabolic acidosis, acute kidney injury with oligoanuria, and left-sided deep vein thrombosis. Despite intensive multidisciplinary interventions, the patient’s repeated refusal of ongoing treatment critically contributed to a fatal outcome. **Conclusions:** This case underscores the high mortality risk associated with opioid–stimulant co-use and the crucial impact of treatment refusal. Clinicians and public health stakeholders should recognize the rapid progression of multisystem dysfunction in polysubstance users and prioritize early, aggressive interventions combined with patient engagement strategies to mitigate fatal outcomes.

## 1. Introduction

“Everywhere, everything, everyone”—this message in the European Drug Report 2024 reflects the rising prevalence of illicit drug use and the limited effectiveness of control measures [1]. Globally, nearly 275 million people have used illicit psychoactive substances or misused pharmaceutical products, excluding alcohol and nicotine, with an 11% projected increase; drug-related deaths in 2019 were ~583,000, exacerbated by COVID-19 disruptions to addiction services [2]. In the EU, preliminary estimates indicate ~7500 drug-related deaths, likely underreported due to incomplete data [3]. Substance use-related morbidity and mortality result from complex interactions of acute and chronic toxic effects and behavioral determinants, with polysubstance use increasingly contributing to severe outcomes [4] (Table 1).

Against this backdrop, the available data delineate a concerning emerging trend—the progressive normalization of polysubstance use. According to the EUDA, opioids are implicated in approximately 70% of overdose-related fatalities, most often in combination with other psychoactive substances. Cocaine is detected in roughly 26% of cases, with its use increasing even in regions where it has traditionally been limited, and increasingly in conjunction with opioids. Other stimulants, including amphetamines and methamphetamines, also contribute to a substantial number of fatal outcomes, again predominantly in the context of combined use with opioids [3]. The presented epidemiological data align with what the National Institutes of Health have termed the “fourth wave of the opioid crisis”, first identified in the United States. Unlike the preceding three phases, which were respectively associated with prescription opioids, heroin, and synthetic opioids such as fentanyl, the current wave is characterized by widespread polysubstance use, wherein opioids are frequently combined with stimulants such as methamphetamine and cocaine. The clinical consequences are multifaceted and highly complex: there is a marked increase in mortality, an expansion of the spectrum of complications, and a diminished effectiveness of standard interventions in emergency toxicology. Concurrently, there is a rising co-morbidity with psychiatric disorders, rendering the fourth wave a challenge not only from a pharmacological standpoint but also from a behavioral perspective [5,6].

Our clinical observations over recent years corroborate the rising trend of polysubstance use. Within the context of emergency toxicology, where life-saving interventions are traditionally focused on critical organs and systems such as the central nervous system, cardiovascular system, and lungs, systemic injuries beyond these primary targets are frequently underestimated, despite their potentially fatal nature [7,8]. To illustrate this issue, we present a clinical case with a fatal outcome, resulting from a complex of complications, including rhabdomyolysis, acute kidney injury, and deep vein thrombosis (DVT), in a patient with concurrent use of fentanyl, cocaine, methamphetamine, and cannabis. To our knowledge, no previously published case reports document this exact combination resulting in such severe multi-system toxicity. This report highlights the extreme risks associated with polysubstance use and provides novel insights into the multifactorial pathogenesis of opioid- and stimulant-induced rhabdomyolysis in the context of acute polysubstance intoxication. The case also illustrates important ethical and legal challenges. The patient initially refused recommended medical interventions. This highlights the complexity of managing acute, life-threatening complications in individuals with substance use disorder.

## 2. Case Presentation

### 2.1. Medical History

A 37-year-old male with a documented history of polysubstance use disorder and chronic Hepatitis C Virus (HCV) infection was admitted to the Emergency Consultative Unit of the Military Medical Academy–Varna. The reason for admission was self-injection of “ten heroin units” (later identified as illicit fentanyl) into the vein of the left foot approximately 16 h prior to hospitalization. The patient also reported concurrent use of methamphetamine and cocaine via inhalation, as well as cannabis via smoking. Following a period of sleep, he experienced pronounced generalized weakness, which was followed by a fall when attempting to rise from bed, accompanied by pain and a sense of stiffness in the left lower leg.

### 2.2. Physical Examination and Clinical Course

The patient was admitted to the Clinical Toxicology Department, Naval Hospital—Varna, Bulgaria Medical Academy, in an acute condition, with inaccessible peripheral venous access; a central venous catheter was therefore established. Upon admission, he was awake and oriented, exhibiting a dysphoric mood and mildly clouded consciousness, with limited insight into his substance use disorder. He reported anuria for more than 24 h. Vital signs were stable, and the patient was able to maintain an active position in bed.

Examination of the skin revealed multiple puncture marks on the left foot, lower legs, and forearms. Bilateral swelling of the hands and fingers was noted. The left lower limb demonstrated pronounced tense edema, with a 3 cm increase in circumference compared to the contralateral limb, accompanied by mild cyanosis and signs of venous congestion. Peripheral pulses were preserved.

An extended panel of laboratory tests was ordered, including muscle and cardiac enzymes, coagulation parameters, arterial blood gas analysis, and electrolyte profiling. Table 2 summarizes the patient’s day-by-day clinical course, highlighting persistence of severe oligoanuria, evolving local vascular complications, and the timing of key therapeutic interventions.

The fluid (volume) balance demonstrated a progressively positive net fluid retention with rapidly worsening oligoanuria (Table 3).

### 2.3. Toxicological Screening and Laboratory Results

Blood and urine samples from the patient were analyzed at the Laboratory of Analytical Toxicology using rapid immunoassay tests (ALL TEST™, MedNet GmbH, Münster, Germany). Due to the available clinical and laboratory data and the clear toxicological profile, additional confirmation by GC–MS or LC–MS/MS was not performed, as such analyses are required only under specific research conditions and were not necessary for clinical management. Negative results in the routine opiate screening prompted additional testing for fentanyl using dedicated mononarcotic immunoassays from the same manufacturer, which yielded a positive result in the urine sample. This finding is fully consistent with the known pharmacokinetic profile of fentanyl: the compound exhibits a short plasma half-life (≈3–8 h), leading to rapid decline of blood concentrations below the detection threshold of routine assays. In contrast, fentanyl and its metabolites are primarily excreted via the kidneys, permitting prolonged detectability in urine, typically up to 24–48 h post-administration [9,10,11]. Furthermore, this case highlights a broader concern: opioid users are sometimes unaware that they are consuming fentanyl, a far more potent opioid, which substantially increases the risk of overdose [12]. Table 4 presents toxicological findings, confirming recent exposure to fentanyl, cocaine, methamphetamine, and cannabis, as detected in urine within 24 h of substance use.

The patient’s laboratory parameters reflect both the severity and dynamic progression of the clinical course and are pathophysiologically consistent with the described case evolution (Table 5). Massive rhabdomyolysis was observed, evidenced by marked elevations in muscle enzymes and related proteins, with progressive increases in biochemical markers of muscle injury. Concurrently, acute kidney injury (AKI) progressed, reflected by rising indicators of renal function corresponding to impaired excretory capacity. Severe hyperkalemia and metabolic acidosis were also documented, indicative of electrolyte imbalance and systemic disruption of acid–base homeostasis, both characteristic of this condition. Urinary protein fractionation was not performed; proteinuria was assessed by semi-quantitative dipstick testing (3+ g/L). Despite the lack of protein stratification, the presence of marked hemoglobin/myoglobinuria (3882 ng/mL) is consistent with pigment-induced tubular kidney injury.

The observed neutrophilia in this patient likely represents a reactive inflammatory response to muscle injury and systemic inflammation associated with rhabdomyolysis. No clinical, microbiological, or radiological evidence of infection was detected, although infection cannot be entirely excluded in immunocompromised patients. Coagulation and vascular assessments identified a DVT, likely precipitated by the combination of inflammation, stasis, and coagulopathy. Due to the absence of arterial access, blood gas analysis was performed on capillary blood obtained from the fingertip. This approach allowed for rapid evaluation of acid–base status and metabolic parameters. Capillary pH, pCO_2_, and HCO_3_^−^ were used for trend monitoring, whereas pO_2_ and oxygen saturation cannot be considered fully arterial. Base excess and lactate provided approximate information regarding metabolic acidosis. Despite these limitations, the analysis clearly demonstrated developing metabolic acidosis associated with AKI and massive rhabdomyolysis.

Serum creatine kinase rose sharply from 63,444 U/L on Day 1 to 161,050 U/L by Day 4, reflecting massive and progressive muscle necrosis. Concurrently, serum creatinine and potassium progressively increased, consistent with worsening acute kidney injury. Hemoglobin/myoglobinuria remained markedly elevated, indicating ongoing pigment-induced tubular injury. These laboratory trends correlate with the patient’s clinical deterioration, demonstrating characteristic patterns of rhabdomyolysis with metabolic derangements and acute kidney injury. Marked deviations from normal ranges highlight the severity of organ dysfunction and the critical multisystem impact despite intensive interventions.

### 2.4. Diagnosis

The patient was diagnosed with polysubstance use disorder (fentanyl, cocaine, methamphetamine, cannabis) and chronic HCV infection. Hospitalization followed intravenous exposure to illicit fentanyl, resulting in severe rhabdomyolysis, AKI with oligoanuria, generalized edema, and left-sided DVT, complicated by multisystem dysfunction. Although a fall was reported, the temporal association with intoxication and the characteristic biochemical profile supported toxin-induced rhabdomyolysis as the principal etiology of muscle pain and edema. Alternative causes were systematically evaluated and excluded. Sepsis or systemic infection were considered unlikely, as the patient was afebrile, inflammatory markers (CRP, procalcitonin) were not suggestive of overt infection, and blood cultures remained negative; antibiotics were administered empirically for prophylactic purposes. Acute arterial thrombosis, arterial dissection, and large-vessel occlusion were ruled out by Doppler imaging, which confirmed venous involvement (phlebothrombosis) without evidence of acute limb ischemia. Clinical and imaging findings were not consistent with acute compartment syndrome requiring fasciotomy. Although prolonged immobilization may have contributed to localized ischemic stress, there was no evidence of primary ischemia–reperfusion injury. Injection-related vascular injury and thrombophlebitis were interpreted as secondary to venous congestion rather than primary drivers of whole-limb ischemia. The potential contribution of administered medications and infusion solutions was considered negligible relative to the toxic effects of illicit drug exposure. Hyperkalemia, oligoanuria, and generalized edema were interpreted as secondary to rhabdomyolysis and consequent renal dysfunction.

### 2.5. Therapeutic Course

The therapeutic course during the patient’s clinical hospitalization is summarized in Table 6 by day. It reflects a multifaceted, multidisciplinary approach, specifically tailored to address the established complex pathophysiology.

## 3. Discussion

The present clinical case illustrates an exceptionally severe polysubstance intoxication involving fentanyl, methamphetamine, cocaine, and cannabis. This resulted in a rare and high-risk toxicological profile. It raises a pivotal question: are the combined effects of these substances merely additive, or do they represent true toxicological synergy that accelerates organ injury? Intranasally administered methamphetamine and cocaine are potent sympathomimetic agents inducing massive catecholamine release, pronounced peripheral vasoconstriction, and a marked increase in skeletal muscle metabolic demand. Consequently, a critical imbalance develops between oxygen delivery and metabolic requirements within skeletal muscle tissue, leading to localized ischemia, mitochondrial dysfunction, and sarcolemmal instability. The clinical manifestation of this cascading pathophysiological process is fulminant rhabdomyolysis [13,14,15,16,17,18,19,20]. Sympathomimetic agents may induce rhabdomyolysis in two ways. First, they exert direct toxic effects on myocytes. Second, they act indirectly through immobilization, mechanical compression, and excessive muscular activity [21]. The involvement of opioids, including fentanyl, in the pathogenesis of rhabdomyolysis is typically multifactorial and may extend beyond classical mechanisms such as prolonged immobilization, muscle compression, or localized ischemia [17,22,23,24,25,26,27,28,29,30]. In the present case, the patient had no documented history of trauma or prolonged immobilization. The rhabdomyolysis was primarily toxin-induced, resulting from the systemic myotoxic effects of fentanyl and other substances. Toxin-related mechanisms include:

○ Direct myotoxicity from fentanyl or adulterants, impairing muscle cell integrity and metabolic regulation [13,14,15];
○ Systemic hypoxic and metabolic stress due to central depressant effects, altered consciousness, and impaired tissue oxygenation;
○ Oxidative and metabolic derangements associated with polysubstance intoxication, enhancing muscle injury [31,32].

Additionally, injection of fentanyl into the left foot likely contributed to locally induced compartment syndrome, evidenced by subfascial edema, neurological deficits, and venous thrombosis, which further potentiated muscle damage. Nevertheless, the primary pathogenesis remains systemic and toxin-mediated rather than trauma-related. This clarification aligns with current toxicology literature emphasizing the multifactorial and context-dependent nature of opioid-associated rhabdomyolysis. Although cannabis is the most commonly used illicit substance worldwide, it has not been convincingly associated with rhabdomyolysis when used in its herbal forms. Available literature suggests that such an association is more characteristic of synthetic cannabinoids, for which cases of rhabdomyolysis and AKI have been reported, albeit rarely as isolated primary etiological factors [33,34,35]. Therefore, in the present case, the role of cannabis is considered likely secondary and modulatory rather than causative or primary.

To systematically assess the potential causality of each substance, we evaluated the temporal relationship between drug administration and symptom onset, the plausibility of dose–response effects, and the exclusion of alternative etiologies. Fentanyl administration preceded the onset of severe rhabdomyolysis and acute kidney injury, consistent with its known myotoxic and systemic effects. Concurrent use of stimulants, including methamphetamine and cocaine, may have further amplified the severity of muscle injury through sympathomimetic-induced hypermetabolism and peripheral vasoconstriction, resulting in a compounded effect on skeletal muscle oxygenation and metabolism. Potential pharmacodynamic and pharmacokinetic interactions were also considered. Opioid-induced central respiratory depression, in combination with stimulant-induced increases in metabolic demand, could accelerate systemic hypoxic and metabolic stress, promoting rapid muscle breakdown. Similarly, local vascular effects, including edema and thrombosis, likely intensified tissue injury. Although cannabis was detected, its contribution is likely modulatory rather than causative, in line with the low incidence of rhabdomyolysis associated with herbal forms of cannabis in the literature. Taken together, these considerations provide a structured mechanistic rationale for the observed clinical severity, supporting the notion that polysubstance exposure may exert additive or synergistic effects on muscle and renal injury. While single-case observations cannot provide definitive quantitative confirmation, the integrated evaluation strengthens the plausibility of the causal link and aligns with established toxicological principles.

**Figure 1:**
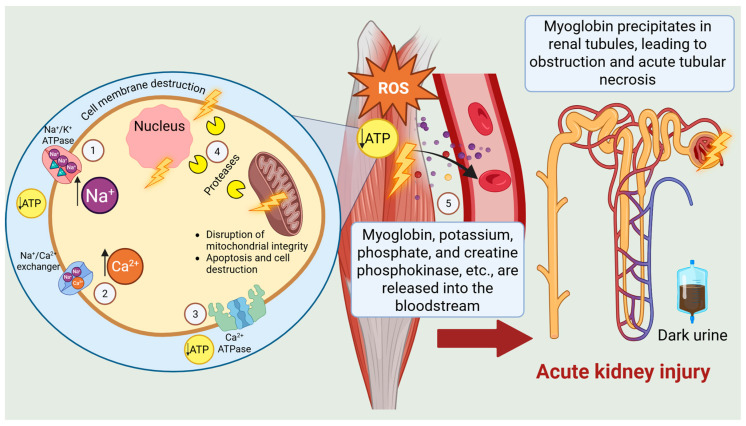
Integrated model of non-traumatic rhabdomyolysis: (1) ATP depletion inhibits Na^+^/K^+^-ATPase activity, resulting in intracellular sodium accumulation. (2) Activation of the Na^+^/Ca^2+^ exchanger increases intracellular calcium levels. (3) Energy deficiency impairs Ca^2+^-ATPase–mediated calcium extrusion; (4) Elevated intracellular Ca^2+^ activates proteases that disrupt plasma and mitochondrial membranes, promoting further Ca^2+^ influx and inducing apoptosis and cell necrosis. (5) Damaged muscle cells subsequently release myoglobin, potassium, phosphate, creatine phosphokinase, and other intracellular constituents into the circulation, causing systemic biochemical disturbances.

The toxicologically induced rhabdomyolysis observed in this patient follows a unified pathophysiological model based on sarcolemma injury and progressive depletion of adenosine triphosphate (ATP) [14,36,37,38,39]. Although ATP depletion is not invariably the initiating event, it almost universally represents the final common pathogenic pathway, culminating in intracellular calcium overload, activation of calcium-dependent proteases, and myocyte necrosis (Figure 1) [15,16,39,40]. Destruction of muscle cells results in massive release of potassium, myoglobin, creatine phosphokinase, and other intracellular constituents into the systemic circulation [36,40,41,42,43,44]. Circulating myoglobin can exceed plasma binding capacity and, under acidic conditions, precipitate in the glomerular filtrate, causing tubular obstruction, necrosis, and renal vasoconstriction [15,36,38,39,42,45,46]. A common complication of rhabdomyolysis is compartment syndrome, where fluid accumulation (up to 10–12 L) in muscle compartments elevates tissue pressure and impairs local perfusion [15,38,41]. Although rhabdomyolysis typically affects the upper limbs and shoulder girdle, muscle injury distribution varies with the causative agent, muscle mass, vascularization, and activity. In this case, the lower limbs were primarily affected, likely due to local susceptibility and repeated intramuscular injections, independent of mechanical trauma [13,14,15,16].

The classic triad of rhabdomyolysis—muscle pain, muscle weakness, and dark-colored urine—was observed in the presented patient. However, it is well established that this combination only occurs in approximately 10% of cases, which, together with etiological heterogeneity, concomitant comorbidities, and behavioral factors, necessitated the application of a more comprehensive diagnostic approach [15]. Serum creatine phosphokinase (CK), established as the most sensitive biochemical marker for skeletal muscle injury, was measured in the patient [14,15,35,43]. Rhabdomyolysis is typically defined by CK levels exceeding 1000 U/L or ≥5 times the upper limit of normal, with concentrations ≥5000 U/L being associated with an increased risk of AKI [14,15,37,41,42,47,48]. In this context, the extreme elevation of CK in our patient (up to 161,050 U/L) reflected massive skeletal muscle injury. The observed increase in serum creatine kinase (CK) during the patient’s treatment was interpreted as part of the natural kinetics of rhabdomyolysis. Peak CK levels are typically reached 48–72 h after the initial muscle injury, even with timely therapy [43]. This delay reflects ongoing CK release from already damaged myocytes. It is further amplified by reduced enzymatic clearance in the setting of acute kidney injury. CK-MB was unreliable for myocardial infarction diagnosis; troponin indicated type 2 myocardial injury. Rapidly progressive AKI developed, with rising creatinine and urea, oliguria progressing to anuria, and urinalysis consistent with tubular injury and myoglobinuria. No clinical signs supported compartment syndrome.

Rhabdomyolysis manifestations range from subclinical enzyme elevations to life-threatening complications, including metabolic acidosis, severe electrolyte disturbances (hyperkalemia, hyperphosphatemia, and hypocalcemia), and AKI. AKI occurs in over 50% of cases, accounting for 5–15% of all acute kidney injury, with 5–26% requiring renal replacement therapy and mortality up to 80% [38,49,50,51]. According to Cabral et al. (2020) [14], dialysis is required in approximately 85% of patients with oliguric AKI and around 30% of those with non-oliguric AKI. Although randomized controlled trials are lacking, available clinical evidence indicates that early and aggressive intervention is associated with a more favorable outcome [14]. In this context, the McMahon score represents a clinically validated tool for the early assessment of the risk of AKI, the need for renal replacement therapy, and mortality upon admission. Despite the absence of serum phosphate data, the McMahon score in our patient remained critically high (≥11 points), reflecting the severity of muscle injury and the elevated risk of dialysis-requiring AKI [14,35,52]. The primary determinants of the high score were extreme elevation of creatine phosphokinase (>160,000 U/L), hyperkalemia, hypocalcemia, and marked increase in serum creatinine. Even with incomplete data, the patient’s elevated McMahon score correlated with persistent oligoanuria, severe hyperkalemia, and multi-organ dysfunction. This confirms the score’s reliability as a prognostic tool and highlights the necessity for early, aggressive interventions. Unfortunately, the patient’s categorical refusal of dialysis in the setting of progressive AKI proved life-threatening and ultimately fatal.

Although xenobiotics—particularly opioids, alcohol, and stimulants—are a leading cause of rhabdomyolysis, clinical studies linking its manifestation to illicit drug use remain limited (Table 7) [31,32,39,43,49,53,54,55].

Many studies lack substance-specific stratification, and none provide robust data on rhabdomyolysis risk from concurrent use of multiple xenobiotics—a scenario relevant to our case. Additional factors, such as viral infections, may further modulate organ vulnerability, complicating the attribution of renal injury to individual substances [21,36,56]. Chronic HCV infection is associated with extrahepatic manifestations, including myalgia and inflammatory myopathies. These conditions may predispose patients to muscle injury and, rarely, rhabdomyolysis, especially in the presence of additional risk factors such as substance abuse or drug interactions [57]. Cases of acute rhabdomyolysis have been reported in patients with chronic HCV infection, either following the initiation of antiviral therapy (e.g., direct-acting antivirals) or in combination with concomitant medications such as statins, where pharmacokinetic interactions can precipitate severe muscle toxicity [58,59]. Moreover, some reported cases among intravenous drug users with HCV indicate that the viral infection may lower the threshold for muscle lysis, thereby facilitating the development of rhabdomyolysis in the context of substance abuse [60]. However, the available evidence is limited and predominantly derived from case reports and observational studies, necessitating cautious interpretation and further investigation. In the present case, although chronic HCV infection may represent a potential vulnerability factor for muscle injury, the available clinical and laboratory findings do not support a direct causal relationship between HCV infection and the fatal outcome observed in this patient.

The patient’s massive rhabdomyolysis caused severe skeletal muscle injury and promoted venous thrombosis. Myoglobin and muscle protein release increased blood viscosity and activated coagulation, while local edema and immobility contributed to venous stasis. Intravenous self-injection into distal lower-limb veins, a high-risk practice among drug users, further predisposes to endothelial injury, thrombo-phlebitis, and proximal thrombus propagation [61,62,63]. The presence of thrombosis involving the popliteal–femoral venous segment was confirmed, supporting the hypothesis of ascending progression of the thrombotic process. Clinically, subfascial edema, limb tension, and cyanotic discoloration were observed, which are typical signs of acute venous obstruction [64]. Although arterial compromise was initially suspected, Doppler imaging revealed no evidence of arterial occlusion, allowing acute arterial thrombosis or embolism to be excluded. The transient attenuation of peripheral pulses was most likely due to extravascular compression from massive edema. The pathogenesis of the thrombosis can be explained via Virchow’s triad: endothelial injury resulted from chemical and mechanical irritation during injection; venous stasis arose from edema and immobilization; and hypercoagulability was driven by systemic inflammation, dehydration, acute renal dysfunction, and a potential hereditary thrombophilia suggested by a family history of DVT [65,66,67]. A diagnostic challenge in this population is the frequent coexistence of chronic venous damage with acute thrombotic episodes, which renders clinical assessment unreliable. In this context, duplex ultrasonography remains the method of choice for differentiating acute DVT from chronic post-thrombotic changes and for excluding arterial pathology [68,69].

Given the patient’s critical condition, intensive intravenous hydration with isotonic solutions (0.9% NaCl, Ringer’s lactate, and 5% Glucose) was initiated to stabilize hemodynamics and correct electrolyte imbalances. Early hydration remains the cornerstone of rhabdomyolysis therapy, despite ongoing debate regarding optimal type, volume, and infusion rate [15,37,42]. Supportive measures included parenteral nutrition, neurometabolic and vitamin supplementation (Piracetam, Thiamine, Pyridoxine, and Cyanocobalamin), and targeted electrolyte correction, while psychomotor agitation was managed with Diazepam and Haloperidol. Due to severe hyperkalemia and progressive oligoanuria, Furosemide was preferred over Mannitol, given the latter’s risks of volume overload, osmotic nephropathy, and hemodynamic instability in renal impairment [35,36,37,38,70,71]. Neither diuretic is proven to prevent rhabdomyolysis-induced AKI in isolation; the primary therapeutic principle remains early, adequate hydration to maintain renal perfusion and reduce tubular myoglobin concentration [21,36,41,72].

Due to the patient’s high thrombotic risk, prophylactic anticoagulation with low-molecular-weight heparin (Enoxaparin) was initiated, followed by continued therapy. The left lower limb was elevated and compression stockings (30–40 mmHg) applied to reduce venous stasis and improve perfusion [73,74,75,76]. Thrombolysis and thrombectomy are generally not recommended in this population due to unreliable histories, superimposed acute on chronic DVT, high bleeding risk, and limited expected benefit [75,76,77,78,79].

Supportive therapy for renal function continued with insulin–glucose solutions, sodium bicarbonate, and calcium as indicated. The use of sodium bicarbonate for AKI prevention remains controversial, with limited evidence [39,72]. Alkalinization may reduce myoglobin toxicity and tubular precipitation, but routine use is not strongly supported [38]. Potential risks include paradoxical intracellular acidosis and volume overload, especially in patients with respiratory or circulatory compromise [42,51].

Despite intensive therapy, persistent oligoanuria and generalized edema continued, with no significant renal improvement. On day five, urgent intermittent hemodialysis was initiated to correct metabolic disturbances while accommodating the patient’s clinical tolerance [80,81,82]. To integrate the temporal sequence of findings and therapeutic decisions, the clinical reasoning in this case can be summarized as follows: the initial presentation, characterized by profound hyperCKemia, progressive AKI, and generalized edema, prompted immediate aggressive fluid resuscitation and initiation of renal supportive measures. Although inflammatory markers were elevated, the absence of fever on repeated assessments and persistently negative blood cultures argued against overt sepsis and favored a systemic inflammatory response secondary to extensive muscle injury. Serial monitoring demonstrated sustained elevation and dynamic changes in serum creatine kinase levels, paralleled by rapid deterioration of renal function indices, which underscored the urgency of considering renal replacement therapy. Despite clear medical indication, the patient’s refusal of dialysis precluded timely implementation, likely contributing to the unfavorable outcome. Taken together, this structured appraisal of evolving laboratory abnormalities, organ dysfunction, and therapeutic decision-making provides valuable insight. It supports the interpretation that severe rhabdomyolytic muscle injury with secondary renal failure was the principal driver of the clinical course in this case. Alternative etiologies were considered but appeared less consistent with the cumulative clinical evidence.

Although a multidisciplinary approach was employed, the patient’s refusal of continued treatment critically influenced the outcome. In individuals with substance use disorders, impaired illness insight, limited recognition of disease severity, and impulsivity may compromise adherence to life-saving therapies [83]. Despite preserved orientation, ongoing intoxication, uremic encephalopathy, and psychiatric consequences of chronic polysubstance use likely impaired risk assessment, leading to discharge with persistent anuria and metabolic derangements. Subsequent multiorgan dysfunction culminated in fatality on day nine post-discharge. No evidence suggested recurrent substance use, indicating that death was primarily due to progression of severe rhabdomyolysis-induced AKI in the context of treatment refusal. Despite extraordinary efforts by the patient’s family to provide support and care, the outcome highlights that urgent and professional medical intervention remains indispensable and potentially life-saving [84,85,86,87]. This case underscores the necessity of early psychiatric involvement, protective measures when ethically justified, and strategies to enhance clinician–patient communication. Systematic evaluation of factors influencing refusal—psychiatric comorbidities, cognitive impairments, prior healthcare experiences, and distrust—remains essential for effective management of high-risk intoxications [4].

This report has several limitations inherent to single-case observations. As an individual clinical case, it cannot establish causal relationships or support generalized epidemiological conclusions. Nevertheless, detailed documentation of such cases provides valuable clinical insights into the complex pathophysiological mechanisms and management challenges associated with severe polysubstance intoxication and its potentially fatal complications.

## 4. Conslusions

The present clinical case demonstrates that the synergistic toxic effects of polysubstance abuse can lead to unpredictable and fatal outcomes, including rhabdomyolysis, AKI, and DVT. Although these complications are less common than central nervous system, cardiac, or pulmonary damage, they are life-threatening and require early recognition and timely therapeutic intervention.

Publishing detailed clinical cases is of critical importance for medical practice and public health, as it provides in-depth toxicological data, an assessment of polysubstance use, and specific mechanisms of toxicity often absent from national registries and official statistics. Such information facilitates the early identification of novel substances, emerging patterns of use, and risk factors, while also enhancing patient prevention and management strategies.

The case further highlights that patient behavior can compromise medical efforts; refusal of therapy led to a fatal outcome despite optimal medical management. This underscores the complex interplay between medical, social, psychological, and legal factors, necessitating an integrated, multidisciplinary approach and specialized care strategies.

In conclusion, this case not only supplements statistical data but also provides critical insight into the risks and dynamics of polysubstance abuse, with direct implications for clinical management, public health, future research, and the development of effective preventive policies. At the same time, it highlights the critical clinical and ethical challenges that arise when patients with life-threatening intoxications refuse life-saving medical treatment.

## Figures and Tables

**Table 1:** Acute and chronic health consequences arising from substance abuse.

Category of Effects	Types
Acute Effects	Intoxication, accidental poisoning, and overdose leading to hospitalization Psychiatric manifestations: anxiety, psychosis, paranoia, acute cognitive impairment Accidents, injuries, or traffic incidents secondary to psychomotor impairment
Chronic Effects	Medical (somatic) morbidity: infectious, pulmonary, metabolic, cardiovascular, and oncological diseases Poor nutrition and hygiene associated with chaotic lifestyle, increasing risk for somatic health problems Psychiatric comorbidity resulting from or exacerbated by substance use

**Table 2:** Clinical timeline.

Day	Clinical Course
Day 1	The patient remained hemodynamically stable, afebrile, alert, and oriented, with persistent pain and edema of the left lower limb. Following urethral catheterization, approximately 200 mL of dark-colored urine, resembling tea, was produced. Urine toxicology screening was positive for psychoactive substances. Serial examinations revealed a livid, markedly edematous, tense, and cool left lower limb with diminished to absent peripheral pulses (+3 cm circumference increase compared to the contralateral limb). Electrocardiogram demonstrated sinus tachycardia with nonspecific repolarization changes. Laboratory findings indicated massive enzymatic release and early signs of multiorgan dysfunction. Cardiological, internal medicine, and surgical consultations were obtained, recommending Doppler ultrasonography and additional vascular assessment. Anticoagulant and symptomatic therapy were initiated. Severe hyperkalemia (K^+^ 8.4 mmol/L) was corrected emergently with pharmacological intervention, diuretics, and intensive laboratory monitoring. Pain and edema persisted without significant change in vascular status by the end of the day.
Day 2	No substantial change in overall condition was noted. The patient remained afebrile, alert, and oriented, with persistent pain and cold edema of the left lower leg, along with generalized edema of the limbs and face. Urine output remained severely reduced (oligoanuria), totaling approximately 200–400 mL in 24 h despite a markedly positive fluid balance. Intensive diuretic and vasoactive therapy was administered without significant effect. Hemodynamics were relatively stable with a tendency toward hypertension. Respiratory status was stable, with no clinical or laboratory evidence of respiratory failure. Laboratory results indicated severe renal impairment (eGFR ≈ 24 mL/min/1.73 m^2^) in the context of ongoing multisystem dysfunction. Extended laboratory tests and consultations with cardiology, nephrology, and vascular surgery were planned for diagnostic clarification and therapeutic planning.
Day 3	The patient spent the night relatively calmly, remaining alert, oriented, and afebrile, with persistent left lower limb pain and generalized edema. Severe oligoanuria persisted (≈200 mL of dark urine in 24 h) despite intensive diuretic and pharmacologic stimulation. Nephrology assessment confirmed acute kidney injury. Hemodynamic and respiratory status remained stable, with a tendency toward hypertension. Vascular surgery consultation revealed tense subfascial edema of the entire left lower limb, neurological deficits in the toes, and reduced active movements. Doppler imaging demonstrated thrombosis of the left popliteo-femoral segment without signs of acute arterial occlusion. Progression to complete anuria was noted. Infusion therapy with vasoactive and diuretic agents was continued without significant clinical effect. Overall condition remained stable, with persistent renal dysfunction, edema, and local vascular complications. Extended laboratory monitoring and multidisciplinary consultations were planned.
Day 4	The patient remained calm, alert, and oriented, with persistent generalized edema of the face, hands, and feet. Severe anuria persisted (≈100 mL/24 h with 900 mL fluid infusion). Renal stimulation therapy with dopamine, furosemide, and novofilin was continued. Despite persistent edema, the patient reported reduced pain, allowing ambulation, without new subjective complaints. Hemodynamically, the patient remained relatively stable (BP: 125–140/68–100 mmHg; HR: 76–90 bpm). Spontaneous breathing and oxygen saturation (95–96%) were normal. Pulmonary and abdominal examinations revealed no pathological findings. In the evening, the patient remained alert, with mild agitation, reporting urethral pain and hematochezia.
Day 5	The patient remained afebrile, with persistent pain in the leg and generalized body pain. The patient was alert, oriented, and cooperative; however, despite institutional restrictions, smoking was observed in the intensive care unit. Urine output was markedly reduced (≈100 mL/24 h). Vesicular breathing was clear. Generalized edema persisted, most pronounced in the left lower limb. Blood pressure remained elevated (up to 175/110 mmHg); symptomatic antihypertensive therapy (chlofazoline) was administered. The patient underwent hemodialysis without complications. Later in the day, he decisively refused continued hospital treatment. He was informed of health and life risks and discharged with accompaniment by his mother.
Day 6	The patient returned to the clinic, accompanied by family insistence. He was informed in detail of the necessity for urgent dialysis and potential complications of treatment refusal. Despite this, he again refused therapy, demonstrating verbal aggression and demanding removal of venous access and urethral catheter. In the presence of on-duty staff and the head of the clinic, he signed a written refusal of treatment. Immediate life-threatening risks were repeatedly explained. The patient was discharged per his insistence with a medical report including recommendations for follow-up and monitoring.
Day 9	The patient died at home following a continued refusal of urgent therapy and hospital treatment.

**Table 3:** Daily fluid balance and urine output during hospitalization.

Hospital Day	Fluid Intake (mL)	Urine Output (mL)	Net Fluid Balance (mL)
Day 1	4600	400	+4200
Day 2	2500	200	+2300
Day 3	900	100	+800
Day 4	700	100	+600
Day 5	Hemodialysis was initiated due to persistent oligoanuria and volume overload		

**Table 4:** Toxicological screening results.

Substance	Cut-Off (Urine, ng/mL)	Cut-Off (Plasma, ng/mL)	Result from the Analysis	
			Urine	Plasma
Amphetamine	1000	80	−	−
Cocaine (Benzoylecgonine)	300	50	+	+
Δ9-Tetrahydrocannabinol metabolite (Marijuana)	50	35	+	+
Benzodiazepines	300	100	−	−
Tricyclic Antidepressants	1000	100	−	−
Barbiturates	300	100	−	−
Morphine (Opiates)	300	40	−	−
Methadone	300	40	−	−
Methamphetamine	1000	70	+	+
3,4-Methylenedioxymethamphetamine	500	50	−	−
Fentanyl	20	15	+	−

**Table 5:** Monitoring of Laboratory Parameters.

Parameter	Reference Range	Day 01	Day 02	Day 03	Day 04	Day 05
Complete Blood Count						
ESR [mm/h]	<20	27.0	–	–	–	–
Hemoglobin [g/L]	130–180	164.0	–	–	126.0	106.0
Erythrocytes [×10^12^/L]	4.8–6.2	5.53	–	–	4.2	3.51
Hematocrit [L/L]	0.35–0.55	0.50	–	–	0.37	0.308
Leukocytes [×10^9^/L]	3.5–10.5	22.21	–	–	14.31	11.1
St [%]	1–6	87.9	–	–	85.8	81.1
Eosinophils [%]	1.5–8	0.0	–	–	0.1	0.0
Basophils [%]	<1	0.01	–	–	0.01	0.0
Lymphocytes [%]	22–50	6.3	–	–	7.2	12.2
Monocytes [%]	2–10	5.7	–	–	5.8	5.7
MCV [fL]	82–100	90.4	–	–	88.1	87.7
MCH [pg]	28–32	29.7	–	–	30.0	30.2
MCHC [g/L]	300–360	328.0	–	–	341.0	344.0
RDW [%]	11.5–14.9	14.2	–	–	14.3	14.4
Platelets [×10^9^/L]	140–440	316.0	–	–	170.0	136.0
MPV [fL]	8.8–12.5	10.8	–	–	10.9	11.3
NRBC [×10^9^/L]	<0.01	0.01	–	–	0.01	0.01
NRBC [%]	0	0.0	–	–	0.1	0.1
Immature Granulocytes [×10^9^/L]	<0.3	0.18	–	–	0.07	0.07
IG [%]	<4	0.8	–	–	0.5	0.6
Neutrophils [×10^9^/L]	2.4–6.9	19.52	–	–	12.27	9.01
Lymphocytes [×10^9^/L]	0.8–3.4	1.39	–	–	1.03	1.35
Monocytes [×10^9^/L]	0.4–1.0	1.27	–	–	0.98	0.74
Biochemistry						
Total Bilirubin [μmol/L]	5–21	7.17	–	–	5.31	7.8
Direct Bilirubin [μmol/L]	<5.13	–	–	–	2.36	1.77
AST [U/L]	<35	**2501.8**	–	–	**2588.8**	**2876.68**
ALT [U/L]	<50	**619.4**	–	–	**826.6**	**887.66**
GGT [U/L]	<55	32.0	–	–	16.3	21.68
α-Amylase [U/L]	28–100	1472.7	–	–	440.6	267.66
Albumin [g/L]	35–53	–	–	–	29.5	28.92
CRP [mg/L]	<5	**14.41**	–	–	**53.08**	**31.02**
Creatine Kinase [U/L]	24–180	**63,444.0**	–	–	**55,050.0**	**161,050.0**
Blood Glucose [mmol/L]	4.1–5.9	5.18	–	–	5.66	5.27
Urea [mmol/L]	2.8–7.2	10.66	–	–	24.05	33.35
Creatinine [μmol/L]	64–104	**260.1**	–	–	**605.2**	**737.0**
Sodium [mmol/L]	135–150	133.0	130.0	128.0	127.0	126.0
Potassium [mmol/L]	3.5–5.5	**8.4**	**6.7**	**6.2**	**6.2**	**6.0**
Chloride [mmol/L]	96–106	100.4	98.9	99.0	98.0	97.0
Total Protein [g/L]	60–83	–	–	–	53.9	53.12
CK-MB [U/L]	<24	8576.8	–	–	2188.9	2360.0
Urinalysis						
pH	4.5–8.0	–	–	–	5.5	–
Specific Gravity	1.010–1.030	–	–	–	1.030	–
Protein [g/L]	0	–	–	–	**3+**	–
Bilirubin	Negative	–	–	–	Negative	–
Urobilinogen [μmol/L]	0–17	–	–	–	Normal	–
Sediment [/μL]	RBC 0–3; WBC 0–5	–	–	–	RBC 34; WBC 32; BACT 13; SQEP 5; UNCC 3	–
Glucose (urine dipstick)	Negative	–	–	–	1+	–
Ketones (urine dipstick) [mmol/L]	Negative	–	–	–	Negative	–
Nitrites [μmol/L]	Negative	–	–	–	Negative	–
Leukocytes (urine dipstick)	0–5	–	–	–	1+	–
Blood in urine	Negative	–	–	–	**1+**	–
Immunology						
Troponin I [ng/L]	<14	**10,375.3**	–	–	–	**2094.66**
Arterial Blood Gas—Capillary						
BE (ecf) [mmol/L]	±3	1.2	–	–	–12.8	–11.6
HCO_3_ act [mmol/L]	22–26	22.1	–	–	12.9	14.1
HCO_3_ stat [mmol/L]	22–26	19.8	–	–	17.0	17.6
O_2_ Sat [%]	95–100	94.6	–	–	84.7	83.8
pCO_2_ [kPa]	4.7–6.0	5.6	–	–	3.3	3.6
pH	7.35–7.45	7.354	–	–	7.34	7.34
pO_2_ [kPa]	10–13	11.2	–	–	6.7	6.6
tCO_2_ [mmol/L]	22–29	24.5	–	–	13.5	14.8
Lactate [mmol/L]	0.5–2.2	–	–	–	1.4315	0.4666
Hemostasis						
Prothrombin Time [s]	11.8–15	21.45	–	16.12	–	15.89
Prothrombin Activity [%]	80–120	49.22	–	–	73.92	68.94
aPTT [sec]	26–38.4	–	–	–	–	36.37
INR	0.7–1.1	1.73	–	–	1.26	1.33
D-dimer [μg/mL]	<0.5	–	–	–	2.97	2.13
Coagulation Screening						
Bleeding Time [sec]	60–180	90	–	–	–	–
Clotting Time [sec]	130–300	210	–	–	–	–

Bold values indicate clinically significant abnormalities related to rhabdomyolysis, acute kidney injury, and systemic toxicity.

**Table 6:** Implemented therapeutic courses and interventions by day.

Day	Therapeutic Goal	Treatment	Dose and Route
1	IV hydration & electrolyte balance	Sodium chloride 0.9% Ringer lactate Glucose 5%	500 mL i.v., 2–4×/day 500 mL i.v., 2–4×/day 500 mL i.v., 4×/day
	Parenteral nutrition	Lipid emulsion	500 mL i.v., 1 bag over 6 h, as needed
	Neurometabolic & vitamin therapy	Piracetam Thiamine Pyridoxine Cyanocobalamin	1 g i.v., 2×/day 3×/day i.v. 3×/day i.v. 1 mg i.m., 1×/day
	Electrolyte correction	Magnesium/Calcium aspartate	1 amp i.v., 1×/day
	Anxiolytic/psychotropic therapy	Diazepam Haloperidol	As needed i.v.
	Anticonvulsant therapy	Carbamazepine	200 mg p.o., 3×/day
	Anticoagulant prophylaxis	Enoxaparin	0.4 mL s.c., 2×/day
	Antibacterial therapy	Ceftriaxone	2 g i.v.
	Metabolic & antioxidant therapy	S-adenosylmethionine	2 × 1 amp i.v.
	Diuretic therapy	Furosemide	1 amp i.v., as needed
	Anti-inflammatory therapy	Dexamethasone	4 mg i.v., 2×/day
2	Diuretic & renal support	Furosemide	2 amp i.v., bolus
	Metabolic/electrolyte correction	Insulin Actrapid + Glucose 10% + Sodium bicarbonate + Calcium gluconate	8E Insulin Actrapid in Glucose 10% 500 mL + 1 amp Sodium bicarbonate + 1 amp Calcium gluconate, 2–3 h i.v. infusion
	Hemodynamic & renal support	Dopamine + Theophylline + Furosemide	Continuous infusion via perfusor
	Anticoagulant therapy	Enoxaparin	0.6 mL s.c., 1×/day
	Venous circulation	Diosmin/Hesperidin	2×/day orally
	Local thrombosis prophylaxis	Heparinoid ointment	100 IU/mg, topical
	Analgesia	Paracetamol	1 fl. i.v.
3	Diuretic & renal support	Furosemide	5–15 amp i.v. + continuous infusion 15 mL/h
	Hemodynamic & renal support	Dopamine + Theophylline	Continuous infusion via perfusor
	Neurometabolic & vitamin therapy	Piracetam Thiamine Pyridoxine	1 g i.v., 2×/day 2×/day i.v. 2×/day i.v.
	Gastroprotection	Pantoprazole	2×/day i.v.
	Antibacterial therapy	Ceftriaxone	2 g i.v.
	Analgesia	Analgin	As needed i.v.
	Anticoagulant prophylaxis	Enoxaparin	0.4 mL s.c., 2×/day
	Venous circulation	Diosmin/Hesperidin	2 × 2 tablets/day
4	Diuretic & renal support	Furosemide	i.v., 10×
	Hemodynamic & renal support	Dopamine Theophylline	1×/day i.v. 1/2 amp i.v., 5×/day
	Correction of metabolic acidosis	Sodium bicarbonate	i.v., 2×/day
5	IV hydration & electrolytes	Sodium chloride 0.9%	100 mL i.v., 2×/day
	Metabolic & renal support	Insulin Actrapid + Glucose 10% + Sodium bicarbonate + Calcium gluconate	8E Insulin Actrapid in Glucose 10% 500 mL + 1 amp Sodium bicarbonate + 1 amp Calcium gluconate, 2–3 h i.v. infusion
	Hemodynamic & renal support	Dopamine + Theophylline + Furosemide	Continuous infusion via perfusor
	Neurometabolic & vitamin therapy	Piracetam Thiamine Pyridoxine	1 g i.v., 2×/day 2×/day i.v. 2×/day i.v.
	Gastroprotection	Pantoprazole	2×/day i.v.
	Antibacterial therapy	Ceftriaxone	2 g i.v.
	Anticoagulant prophylaxis	Enoxaparin	0.4 mL s.c., 2×/day
	Hypertension control	Clonidine	0.15 mg, tablets
	Renal support	Hemodialysis	–

**Table 7:** Incidence of rhabdomyolysis and fatal outcomes associated with individual psychoactive substances and alcohol in clinical and epidemiological studies, presented chronologically.

Source	Study Design	Number of Patients with Rhabdomyolysis Due to Substance Use	Rhabdomyolysis (%) by Substance	Fatality (%) by Substance
Welte T. (2004) [53]	Retrospective forensic study	103 (drug deaths)	Heroin/other opioids—NR Methadone—NR Cocaine—NR Alcohol—NR Benzodiazepines—NR	50.5% of fatal drug abuse cases showed confirmed or probable rhabdomyolysis based on the presence of myoglobin in renal tissue (no stratification by substance)
Rodríguez E. (2013) [55]	Retrospective cohort	35 (of 126)	Heroin—24% Cocaine—22.4% Other substances—19.8% Alcohol—13.5% “Smart drugs”—5.6%	NR
Lau Hing Yim C. (2019) [49]	Retrospective cohort	77 (of 643)	NR	NR
Waldman W. (2021) [43]	Observational (Euro-DEN)	468 (of 1015)	Cocaine—22.9% Amphetamine—16.2% Cannabis—15.8% GHB/GBL—15.4% Heroin—14.3%	NR
Amanollahi A. (2023) [31]	Systematic review & meta-analysis	NR	Heroin—57.2%^†^ Amphetamines—30.5% ^†^ Methamphetamine—40.3% ^†^ MDMA—19.9% ^†^ Cocaine—26.6% ^†^ Tramadol—17.1% ^†^ Methadone—16.1% ^†^ Synthetic cannabinoids—10.3% ^†^ Opioids overall—8.8% ^†^ Ethanol—3.0% ^†^ Methanol—2.0% ^†^	NR
Eghbali F. (2025) [32]	Cross-sectional clinical	455 (of 788)	Methadone—41.5% Benzodiazepines—10.1% Opium—6.1%	Methadone—5.2% Benzodiazepines—0.8% Others—NR

NR = Not reported; ^†^ Substance-specific incidence proportions across studies.

## Data Availability

The original contributions presented in this study are included in the article. Further inquiries can be directed to the corresponding author.

## Abbreviations

The following abbreviations are used in this manuscript:

AKI	Acute kidney injury
ATP	Adenosine triphosphate
CK	Creatine phosphokinase
DVT	Deep vein thrombosis
EUDA	European Union Drugs Agency
HCV	Hepatitis C Virus
